# SDH5 Depletion Enhances Radiosensitivity by Regulating p53: A New Method for Noninvasive Prediction of Radiotherapy Response

**DOI:** 10.7150/thno.34443

**Published:** 2019-08-14

**Authors:** Yan Zong, Qianwen Li, Furong Zhang, Xunde Xian, Sihua Wang, Jiahong Xia, Jie Li, Zhan Tuo, Guangqin Xiao, Li Liu, Guiling Li, Sheng Zhang, Gang Wu, Jun Liu

**Affiliations:** 1 Cancer Center, Union Hospital, Tongji Medical College, Huazhong University of Science and Technology, Wuhan 430022, China; 2 Department of Molecular Genetics, University of Texas Southwestern Medical Center, Dallas, United States; 3 Department of Cardiac Surgery, Union Hospital, Tongji Medical College, Huazhong University of Science and Technology, Wuhan 430022, China

**Keywords:** SDH5, p53, Radiotherapy, Apoptosis, Lung cancer, Noninvasive, Prediction

## Abstract

Radiotherapy is an effective treatment for lung cancer but lacks a reliable prediction method. Cell-free nucleic acids in plasma have been reported as a novel tumor marker. Here, we evaluate circulating succinate dehydrogenase 5 (SDH5) mRNA in plasma and SDH5 protein in tumors, assess their predictive value in lung cancer patients undergoing radiotherapy, and explore the underlying mechanisms.

**Methods**: SDH5 expression was measured in peripheral blood samples and fresh tumor specimens from 208 non-small cell lung cancer (NSCLC) patients and correlated with clinical outcomes. SDH5 knockout mice and human xenograft mice were used to evaluate radiosensitivity. Cell growth, apoptosis, and the DNA damage response were assessed. Relevant RNA and protein levels were analyzed by qRT-PCR and Western blotting. Immunoprecipitation and GST pulldown assays were performed to detect protein-protein interactions. Polyubiquitination of p53 was examined by an in vitro ubiquitination assay.

**Results**: Plasma and tumor SDH5 mRNA levels were positively correlated (rho=0.894, P<0.001). Patients with relatively low SDH5 levels in plasma (0.47, 0.12-0.89) and tumors (3.85, 0.96-7.23) had a better prognosis after radiotherapy (median PFS: 30.0 versus 15.0 months, hazard ratio: 0.276, 95% CI: 0.201-0.379, P<0.001). In SDH5 knockout mice, the lung epithelial cells exhibited increased DNA damage after radiation. In human lung xenograft mice, SDH5-deficient tumors had a smaller volume after radiotherapy. Furthermore, SDH5 depletion inhibits p53 degradation via the ubiquitin/proteasome pathway, which promotes apoptosis and enhances radiosensitivity in NSCLC.

**Conclusion**: Our findings provide a novel noninvasive method for prediction of response to radiotherapy and may have significant implications for cancer radiotherapy.

## Introduction

Globally, lung cancer has the highest incidence and mortality among cancers in men and the third highest incidence and second highest mortality among cancers in women 1. According to estimates, there were approximately 14.1 million new cancer cases worldwide in 2012, and approximately 1.56 million people died of lung cancer, accounting for nearly twenty percent of cancer-related deaths 1, 2. Of all lung cancers, approximately 85% of cases are non-small cell lung cancer (NSCLC). Moreover, in advanced NSCLC, radiotherapy (RT) is among the most important therapies that can improve the average survival time and quality of life 3**.** However, a subgroup of NSCLC patients shows low sensitivity to RT and progresses rapidly 4, 5. Therefore, determining which subsets of patients are sensitive to RT is critical.

The recent development of molecular biology and genetics has contributed to the subclassification of tumors, which can be used to determine prognosis or perform assays to predict response to RT 6. For example, p53 is a master tumor suppressor with several downstream target genes (such as p21) 7. Numerous studies have shown that p53-mediated apoptosis is among the main mechanisms of RT for tumors. Indeed, our previous study indicated that CDK16 in tumor tissue promotes radioresistance by modulating the p53 signaling pathway 8. However, lung biopsy is an expensive procedure with a risk of several complications 9, 10 and intrinsic sampling variability because of the small sample size 11. Moreover, this approach is unacceptable for patients who cannot tolerate biopsy. Therefore, the development of reliable noninvasive methods for the prediction of the RT response is essential for guiding lung cancer therapy.

In the field of oncology, biomarkers have significant diagnostic, prognostic and predictive value, and highly sensitive and specific markers are particularly important for clinical precision medicine. Although serum tumor markers, such as carcinoembryonic antigen (CEA), neuron-specific enolase (NSE), and squamous cell carcinoma antigen (SCC-Ag), have been widely used as convenient diagnostic markers, their lack of sufficient sensitivity and specificity in the early detection of lung cancer has limited their application 12, 13. Accumulated evidence has shown that tumor-associated circulating cell-free mRNA in plasma or serum is increased in cancer patients 14-16. Compared to protein markers, RNA markers are more sensitive and specific. Furthermore, the detection cost of RNA markers is much lower than that of protein markers; thus, these circulating cell-free mRNAs are considered new noninvasive biomarkers for diagnosing disease or predicting prognosis.

Succinate dehydrogenase 5 (SDH5), which is also known as SDHAF2 and required for the flavination of succinate dehydrogenase, has been reported to contribute to the development of several types of cancers 17-19. Previously, we demonstrated that loss of SDH5 could facilitate EMT and lead to lung cancer metastasis via the glycogen synthase kinase 3β/β-catenin pathway 20. In the present study, we found not only that the SDH5 protein can be detected in tumors but also that SDH5 mRNA can be detected in plasma by qRT-PCR, indicating its predictive effect in RT. Our data further show that SDH5 modulates radiosensitivity by directly binding p53 and promoting phosphorylation of cytoplasmic p53 at Ser315. This phosphorylation ultimately accelerates p53 degradation via the ubiquitin/proteasome pathway and influences radiosensitivity. These findings reveal a novel noninvasive method for the prediction of the RT response and an important new regulatory mechanism of p53.

## Methods

### Clinical specimens

Human lung cancer and paracancerous lung tissue samples were obtained from Wuhan Union Hospital. The blood samples were collected within a week prior to receiving RT. All samples were anonymized. All protocols using human samples were reviewed and approved by the Ethical Committee of Huazhong University of Science and Technology. Written informed consent was obtained from all patients.

### Study design

We initiated an analytical, observational, open, and retrospective study (ChiCTR1800014878) involving 208 patients with stage III lung cancer who were ready to receive RT. The patients received concomitant chemotherapy with TP or EP. The radiation dose was 60-66 Gy in 30-33 fractions, 2.0-2.2 Gy per fraction, 1 fraction per day. The short-term response to the RT treatment was evaluated by chest computed tomography (CT) imaging at 1 month after RT.

### Cell culture

The cell lines (including A549, HCC-827, NCI-H292, NCI-H226, NCI-H460, NCI-H522, HCT116 P53(-/-), HCT116 P53(+/+), and HEK293t) were purchased from the Chinese Academy of Science Committee on Type Culture Cell Bank (Shanghai, China) and cultured in RPMI 1640 (Gibco, USA) or DMEM (Gibco, USA) supplemented with 10% fetal bovine serum (FBS, Gibco, USA).

### Regents and antibodies

MG132 was purchased from Selleckchem. Cycloheximide was purchased from Calbiochem. Phos-tag acrylamide was purchased from Wako. The TIAamp Virus RNA kit obtained from Tiangen was used to extract RNA from plasma. The following primary antibodies were used: anti-SDH5 (Cell Signaling Technology, USA), anti-P53 (Santa Cruz Biotechnology, USA), anti-MDM2 (Abcam, UK), anti-P21 (Cell Signaling Technology, USA), anti-γH2AX (Cell Signaling Technology, USA), anti-H2AX (Cell Signaling Technology, USA), anti-Noxa (Cell Signaling Technology, USA), anti-BCL2 (Cell Signaling Technology, USA), anti-BAX (Cell Signaling Technology, USA), anti-PUMA (Cell Signaling Technology, USA), anti-Caspase9 (Cell Signaling Technology, USA), anti-Cleaved Caspase 9 (Cell Signaling Technology, USA), anti-Caspase3 (Cell Signaling Technology, USA), anti-Cleaved Caspase3 (Cell Signaling Technology, USA), anti-DNA-PKcsThr2609 (ABclonal Biotech Co., China), anti-DNA-PKcs (ABclonal Biotech Co., China), anti-ku70 (ABclonal Biotech Co., China), anti-ku86 (ABclonal Biotech Co., China), anti-HA (Cell Signaling Technology, USA), anti-flag (Santa Cruz Biotechnology, USA), anti-GAPDH (Abcam, UK),anti-Actin (Cell Signaling Technology, USA), and anti-laminA (Santa Cruz Biotechnology, USA).

### SiRNA transfection

siRNA targeting SDH5 was purchased from Santa Cruz Biotechnology (sc-96879), with the following sequences: Control-SiRNA: 5'-UUCUCCGAACGUGUCACGUtt-3'; SDH5-SiRNA1: 5'-CCAAGUGUACUCAAAGAAAtt-3'; and SDH5-SiRNA2: 5'-GGAUGGUAACUACUUAUGAtt-3'. Cells were transfected with 50 nM siRNA. GenMute transfection reagent (SL100568, SignaGen Laboratories, China) was used according to the manufacturer's instructions.

### Western blot analysis

Cells were lysed in RIPA buffer. Equal concentrations of total proteins were separated on SDS-PAGE and transferred to PVDF membranes (Millipore, USA). After blocking with 5% skim milk, the membranes were incubated with primary antibodies overnight at 4°C. The membranes were washed with Tris-buffered saline Tween (TBST) buffer and incubated with secondary antibodies (Invitrogen, USA) for 1 h at room temperature, and proteins were visualized using an enhanced chemiluminescence kit (Santa Cruz Biotechnology, USA).

### Cytoplasmic and nuclear protein fractionation

Cells were transfected with scramble or SDH5-siRNA. After 48 h, the cells were collected. Cytoplasmic and nuclear fractions were separated using an NE-PER Nuclear Cytoplasmic Extraction Reagent kit (Thermo Fisher Scientific) according to the manufacturer's procedures.

### In vitro ubiquitination assay

Cells were transfected with the indicated siRNAs or constructs. MG132 (10 µM) was added 4 h before harvesting. Then, the cells were lysed with RIPA buffer. The supernatants were incubated with protein A/G (Millipore, CA, USA) and the indicated antibody overnight at 4°C and analyzed by Western blotting.

### Immunohistochemistry

Tumor tissues were fixed in Formalin, embedded with paraffin and cut into sections. Then, the tissues were deparaffinized, rehydrated and stained with primary antibodies overnight at 4°C. After incubating with secondary antibodies (Boster, Wuhan, China) for 1 h and counterstaining with hematoxylin for 30 s, the sections were visualized under a light microscope.

### Plasma RNA extraction

Peripheral venous blood samples (3 ml) were collected from patients who were ready for RT into tubes containing ethylenediaminetetraacetic acid (EDTA). Then, the samples were centrifuged within 2 h at 4000 rpm for 20 min at 4°C. In total, 500 µl plasma from each sample were subjected to RNA extraction following the manufacturer's instructions. After RNA extraction and purification, quantitative RT-PCR was used to detect expression of GAPDH (as an endogenous control) and the genes of interest. Each batch of reaction included a positive control from commercial human lung RNA (Stratagene, La Jolla, CA, USA) as calibrators.

### Quantitative RT-PCR

Total RNA was extracted using TRIzol Reagent (Invitrogen). SYBR® Premix Ex Taq™ II (Takara Bio, Japan) was used to reverse-transcribe the total RNA into cDNA according to the manufacturer's instructions. The amplification was performed using SYBR-Green Master Mix (Takara Bio, Japan) with a Step One Plus Real-Time PCR system (Applied Biosystems). The following sequences were used: SDH5 forward primer 5'-GACTTCGTCGCTGATGCTTG-3' and reverse primer 5'-GTTGGGCTGTCACCTCTGTA-3'; P53 forward primer 5'-CTCCTCAGCATCTTATCCGAGTG-3' and reverse primer 5'-GTGGTACAGTCAGAGCCAACC-3'; P21 forward primer and reverse primer; MDM2 forward primer 5'-AGGTGGACCTGGAGACTCTCA-3' and reverse primer 5'-CGGCGTTTGGAGTGGTAGAAA-3'; and GAPDH forward primer 5'-ACCACAGTCCATGCCATCAC-3' and reverse primer 5'-TCCACCACCCTGTTGCTGTA-3'. Triplicate runs of each sample were normalized to GAPDH mRNA to determine relative expression.

### Co-Immunoprecipitation

Cells were lysed in RIPA buffer, and the supernatants were incubated with protein A/G (Millipore, CA, USA) and the indicated antibody overnight at 4°C. After centrifugation at 3000 rpm for 3 min, the precipitates were washed 3 times with RIPA buffer. Then, the samples were analyzed by Western blotting.

### GST pull down assay

GST-vector or GST-P53 fusion proteins purified from bacteria were immobilized on GST beads (GE Healthcare) and incubated with lysates prepared from HEK293T cells transiently transfected with Flag-tagged SDH5 for 2 h at 4 °C. The samples were washed five times and analyzed by Western blotting.

### Lentiviral infection

SDH5 shRNA lentiviral particles were purchased from Santa Cruz Biotechnology (sc-96879-V). Monolayer cells were transfected according to the manufacturer's instructions. Cells expressing SDH5 shRNA were screened with medium containing 1 µn/ml puromycin.

### Cell viability assay

Tumor cells were cultured in 96-well plates at 5X103 cells per well and allowed to adhere overnight. The cells were transfected with 50 nM Control-SiRNA or SDH5-SiRNA for 48 h, and then, these cells were exposed to the indicated doses of radiation using a linear accelerator on 0.5 cm compensation by 6 MV Xrays. Cell viability was measured by a CCK-8 kit (sigma) according to the manufacturer's instructions. All experiments were performed using 6-8 wells per experiment and repeated at least three times.

### Animal experiments

Animal experiments were reviewed and approved by the Ethical Committee of HUST. For the orthotopic mouse model of lung cancer, the lungs of male nude mice (6-8 weeks of age; n=3 per group) were exposed and injected with 5X10^5^ cells suspended in 20 µl of phosphate-buffered saline (PBS). One week after the injection, the surgical staples were removed, and the tumors were planted in lung tissue and imaged by bioluminescent imaging (BIL; Xenogen). Then, the tumors were exposed to 0 and 20 Gy of X-ray radiation and monitored by bioluminescent imaging (Lago X, Cold Spring Biotech Corp.; 20s exposure). The indicated proteins were measured by Western blotting. For the SDH5 knock-out mouse model, we previously reported the KO strategy 20. The lungs of SDH5 knock-out mice (n=3 per group) were exposed to 0 and 15 Gy of X-ray radiation, and then, the indicated proteins were measured by Western blotting. Furthermore, for orthotopic mouse model of subcutaneous tumor was established as previously mentioned; briefly, 1X106 KD (SDH5 knock down) or Con (SDH5 con-expressing) HCT116 p53 (+/+) and HCT116 p53 (-/-) cells were subcutaneously injected in athymic nude mice (n=3), and the tumors received 40 Gy radiation (8 Gy/day *5 days). The tumor growth and indicated proteins in the tumors were measured.

### Statistical analysis

The cut-off value of SDH5 expression was determined by X-tile software 21. Progression-free survival (PFS) and overall survival (OS) were estimated using the Kaplan-Meier method and compared with a nonparametric log-rank test. All experiments were performed at least 3 times. The bars represent the mean ± SD. The statistical analyses were performed using SPSS version 23. P<0.05 was considered significant.

## Results

### SDH5 can be detected not only in tumor tissue but also in plasma, and its expression is associated with the RT response

**Figure 1:**
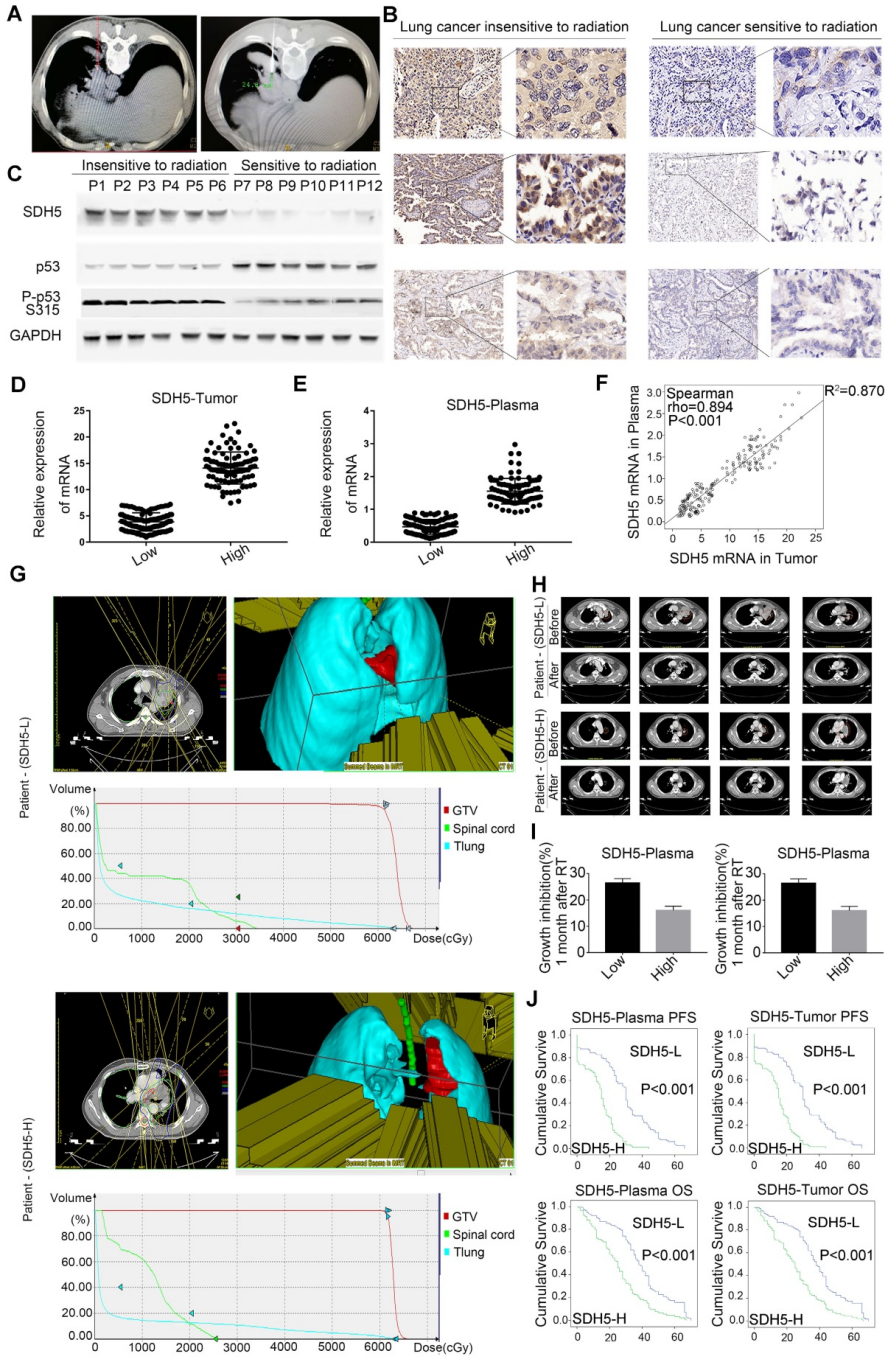
** SDH5 expression levels in plasma and tumors were positively correlated and could predict the RT response. (A)** Representative images of CT-guided percutaneous needle biopsy of lung cancer. **(B)** IHC staining of SDH5 expression in tumors sensitive or resistant to radiation (magnification, 400 x). **(C)** Immunoblotting analysis of SDH5 and p53 in specimens from lung cancer patients before radiation. **(D)** mRNA levels of SDH5 in plasma samples from lung cancer patients before radiation. **(E)** mRNA levels of SDH5 in tumor samples from lung cancer patients before radiation. **(F)** Correlation between plasma and tumor mRNA expression of SDH5 (n=208). **(G)** In patients with different SDH5 expression levels (low or high), radiation for lung cancer was administered by focusing on 5 directions. Axial view, 3D image of radiation dose distribution, and average dose-volume histogram of the GTV and organs at risk. **(H)** Representative computed tomography (CT) images of irradiated lesions 1 month after radiation from 2 patients with different SDH5 expression levels. **(I)** Measurement of the changes in the tumor volume before and 1 month after radiation in patients with different SDH5 expression levels (detected in plasma and the tumor; low expression in 115 patients, high expression in 93 patients). **(J)** Kaplan-Meier analysis of progression-free survival and overall survival in lung cancer patients stratified by SDH5 expression (detected in plasma and the tumor). The data are presented as the mean ± s.d. GAPDH was used as a loading control for the immunoblotting analysis (P1-P12: Patient 1-Patient 12).

To examine the relationship between SDH5 and the RT response, we initiated an analytical, observational, open, retrospective study (ChiCTR1800014878) involving 208 patients with stage III lung cancer who were ready to receive RT. The characteristics of all patients are shown in Table 1. Most patients were males (58.65%), and the predominant histology was squamous carcinoma (75.48%). In total, 176 (84.62%) patients had stage IIIB disease. The mRNA expression levels in plasma were stable after incubation at room temperature or on ice for 30 min, 1 h, 1.5 h or 2 h (Figure S1). Blood and tumor samples were collected before treatment to detect the SDH5 concentrations. The specimens were obtained from the lung cancer patients before RT via CT-guided percutaneous needle biopsy (Figure 1A). As shown in Figure 1B-C, SDH5 was expressed in both the cytoplasm and nucleus as confirmed by immunohistochemistry (IHC) and Western blotting. Moreover, those patients with tumors that were significantly smaller one month after RT had a lower expression of SDH5 (Figure 1B), and the loss of SDH5 expression was correlated with the upregulation of p53, which is the most extensively studied tumor suppressor gene, in the clinical specimens from the lung cancer patients (Figure 1C). p53 is regarded as a potential target for RT because of its critical role in determining radiosensitivity by modulating apoptosis, cell cycle redistribution and DNA damage. More importantly, SDH5 can be detected in plasma by qRT-PCR, and the Spearman rank correlation test revealed a correlation between the plasma and tumor SDH5 mRNA levels (rho=0.894, P<0.001) (Figure 1D-F). Therefore, we speculated that SDH5 expression in plasma is associated with the RT response in NSCLC patients.

To further study the relationship between SDH5 expression and the response of patients to RT, irradiated lesions were evaluated by computed tomography (CT) imaging using the response evaluation criteria in solid tumors (RECIST). As shown in Figure 1G, among patients with different SDH5 expression levels, 3D irradiation was administered from a total of 5 fields, and the average delivered dose-volume histograms of the gross tumor volume (GTV) and organs at risk were similar, but the tumor shrinkage one month after RT showed an obvious difference (Figure 1H). Then, we calculated the changes in the tumor volume before and after RT in the patients with different SDH5 expression levels, followed by normalization based on the tumor volume with the level before radiation set as 1. As shown in Figure 1I, the tumors from the patients with lower SDH5 expression in either plasma (0.47, 0.12-0.89) or the tumor (3.85, 0.96-7.23) shrank rapidly. More importantly, the patients with SDH5 deficiency had better survival (average median progression-free survival (PFS): 30.0 versus 15.0 months, hazard ratio: 0.276, 95% confidence interval: 0.201-0.379, log-rank test, P<0.001; average median overall survival (OS) 38.0 versus 25.0 months, hazard ratio: 0.471, 95% confidence interval: 0.355-0.626, log-rank test, P<0.001), and the results from the plasma and tumor samples were consistent (Figure 1J). Taken together, these results indicate that SDH5 detection in plasma may be a new noninvasive method for the prediction of the RT response in NSCLC patients.

### Knockdown of SDH5 enhanced radiosensitivity in lung cancer cells

**Figure 2:**
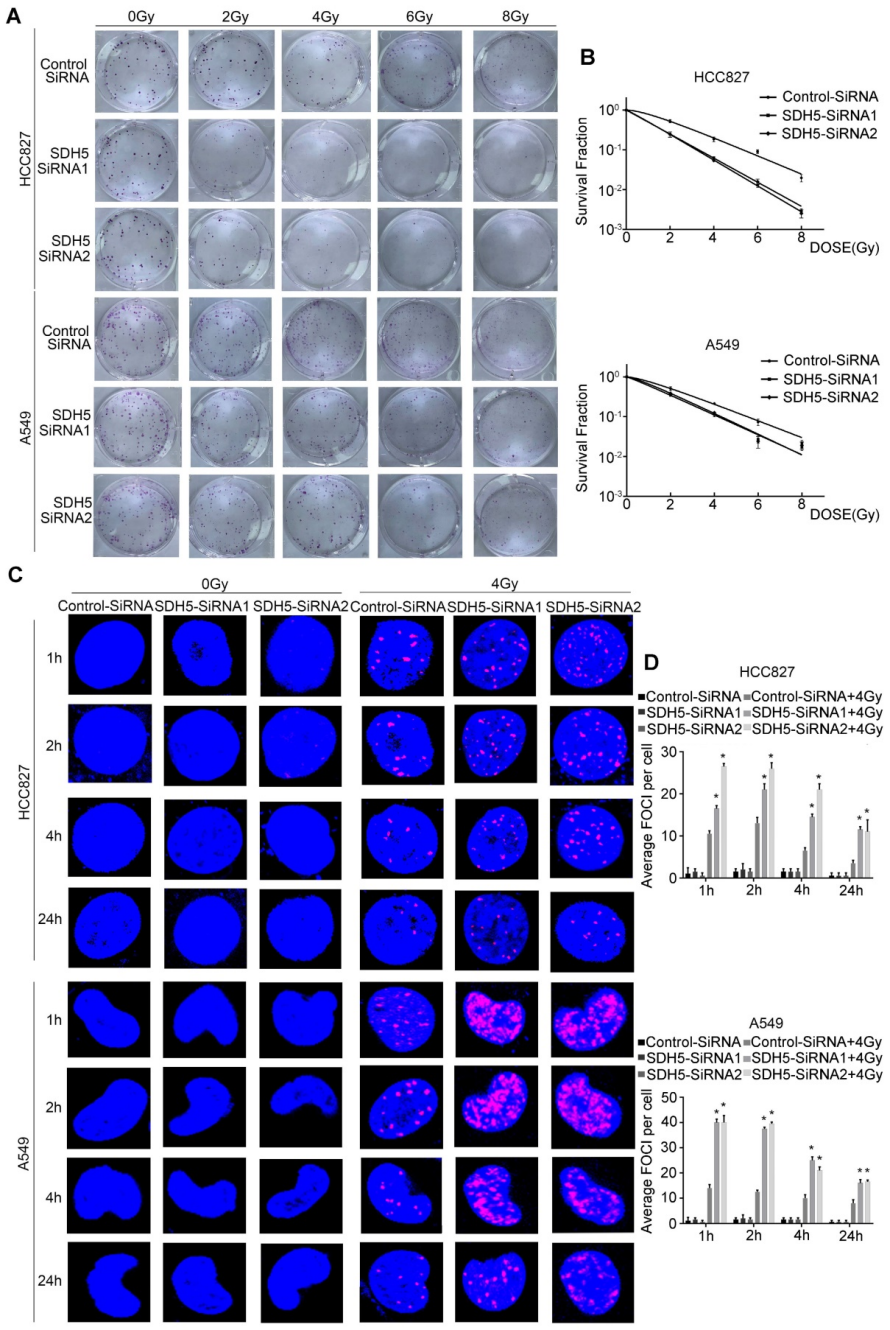
** SDH5 silencing results in enhanced radiosensitivity in lung cancer cells**. **(A)** Representative images of colony formation in HCC827 and A549 cells pretreated with SDH5-SiRNA or Control-SiRNA for 48 h and then exposed to 2, 4, 6 or 8 Gy of X-ray irradiation. **(B)** Clonogenic cell survival curves were generated for HCC827 cells treated with SDH5-SiRNA or Control-SiRNA after radiation. The survival data were normalized to those of the unirradiated control cells. **(C)** Representative images of the γ-H2AX foci (γ-H2AX is labeled in red, and the nuclei are labeled in blue). A549 and HCC827 cells were pretreated with SDH5-SiRNA or Control-SiRNA for 48 h and exposed to 4 Gy of X-ray irradiation, followed by immunofluorescence staining. **(D)** The average number of γ-H2AX foci per cell was significantly increased after 4 Gy irradiation in the SDH5-deficient cells. The results are shown as the mean ± SD of at least three independent experiments. *P<0.05 compared to the radiation alone group.

**Figure 3:**
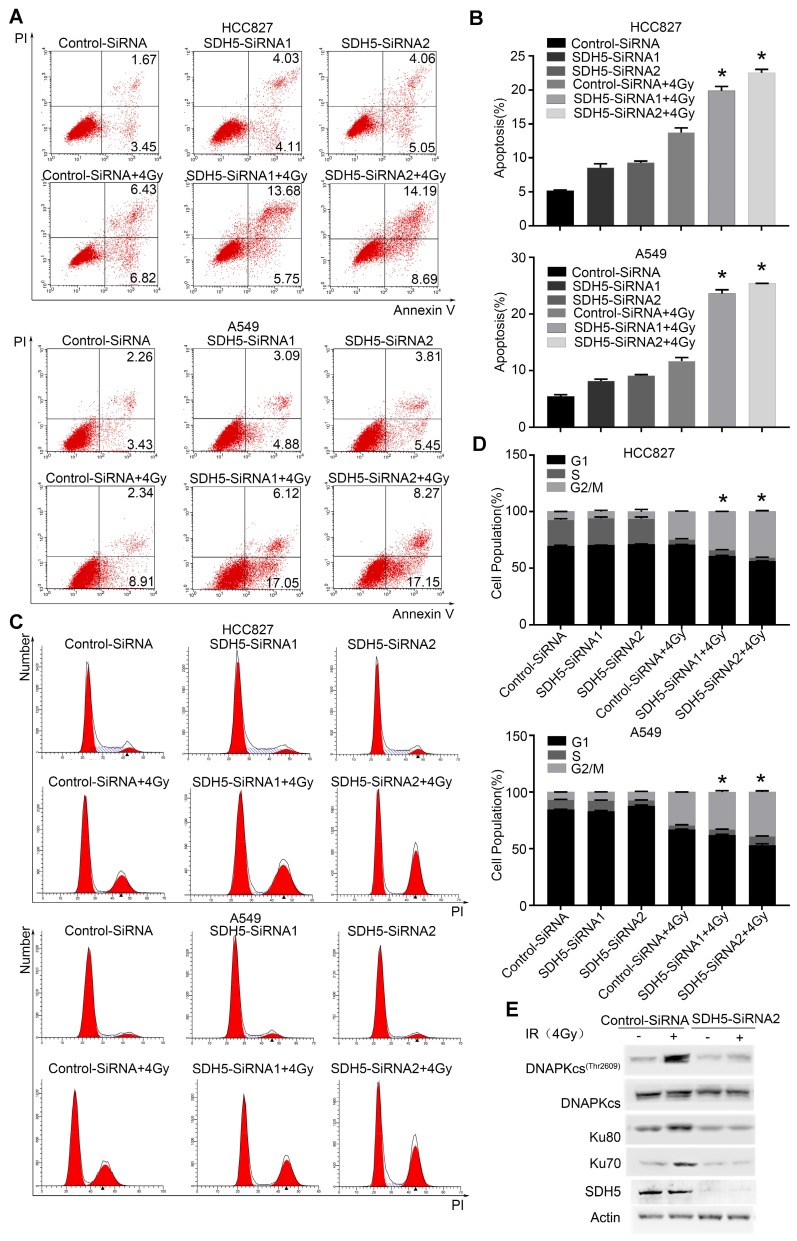
** SDH5 silencing promotes apoptosis and G2/M cell cycle arrest in NSCLC after radiation. (A)** Apoptosis was remarkably increased in the SDH5-deficient HCC827 and A549 cells. Cells were transfected with the indicated siRNAs. After 48 h, the cells were subjected to annexin V-EGFP/propidium iodide staining and analyzed by flow cytometry. **(B)** Percentages of total annexin V-positive cells after SDH5 silencing (n=3). **(C)** Cell cycle distribution of SDH5-deficient HCC827 and A549 cells after radiation. Cells were transfected with the indicated siRNAs for 48 h, and the cells were exposed to 4 Gy of X-ray irradiation, followed by PI staining and a flow cytometric analysis. **(D)** Percentages of each cell cycle phase per group (n=3). **(E)** Immunoblotting analysis of the indicated proteins in A549 after 4 Gy radiation. The data are presented as the mean ± s.d. Actin was used as a loading control for the immunoblotting analysis.

Our clinical data suggest that SDH5 downregulation may enhance radiosensitivity in patients with lung cancer. To confirm the functional effects of SDH5 knockdown on radiosensitivity, a clonogenic assay and immunofluorescence staining for γ-H2AX were performed in vitro. As shown in Figure 2A-B, the HCC827 cell line and A549 cell line, transfected with SDH5-SiRNA were relatively sensitive to irradiation in the cell survival curve analysis. SDH5 deficiency significantly increased the number of γ-H2AX foci per cell at both 1 h and 24 h after 2 Gy radiation in the A549 and HCC827 cell lines (Figure 2C and 2D). Detection of apoptosis and the cell cycle showed that decreased expression of SDH5 resulted in an obvious increase in apoptosis (Figure 3A-B) and that the cells were arrested in the G2/M phase (Figure 3C-D). Although many scientists believe that G2 arrest can allow cells more time to repair DSBs after RT, this finding appeared paradoxical in this case. However, the level of γ-H2AX at 24 h was consistently higher in the combination group than in the radiation alone group, suggesting that extended G2 arrest likely resulting from the prolonged process of DNA damage repair could also contribute to SDH5 deficiency-mediated radiosensitization. Moreover, molecular pathway analysis showed that loss of SDH5 expression was correlated with downregulation of DNA-PKcs (Thr2609) and ku86, which play important roles in DNA repair (Figure 3E and Figure S2). Ku86 and DNA-PKcs are the key components of the DNA-dependent protein kinase (DNA-PK) complex, which is involved in DNA double-strand break repair by nonhomologous end joining (NHEJ). These results suggest that the SDH5 deficiency increased the radiosensitivity of NSCLC cells in vitro.

### SDH5-deficient tumors in orthotopic mice and lung epithelial cells from SDH5 knockout mice both exhibit increased radiosensitivity due to p53 activation

**Figure 4:**
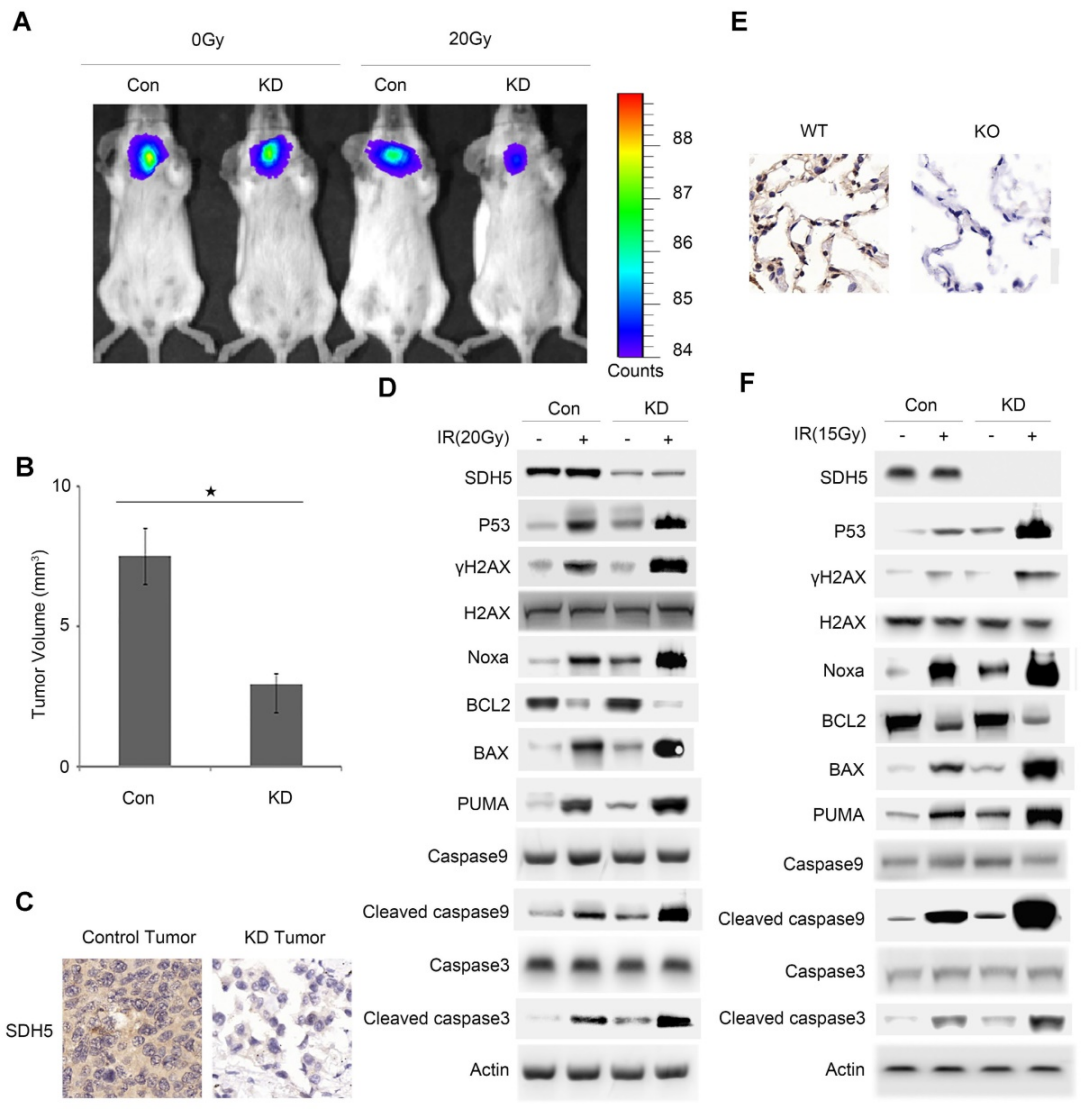
** Deficiency of SDH5 increases radiosensitivity in both orthotopic mice and SDH5 knockout mice by activating p53. (A)** Representative BIL images of mice bearing A549-KD tumors after radiation. Mice (n=3) were imaged after 10 days to determine the local tumor growth. **(B)** Histograms of the quantification of the volume of the Con or KD tumors at the same time after radiation. ★P<0.05 (n=3 per group). **(C)** Expression of SDH5 in paraffin-embedded tumor sections (magnification, 400x). **(D)** Immunoblotting analysis of various proteins associated with radiosensitivity and apoptosis in tumors after radiation. **(E)** IHC staining of lung tissues from wild-type and SDH5 knockout mice (magnification, 400x). **(F)** Immunoblotting analysis of the indicated proteins in lung tissues from wild-type and SDH5 knockout mice 10 days after 15 Gy/10F radiation. The data are presented as the mean ± s.d. Actin was used as a loading control for the immunoblotting analysis (Con: SDH5 con-expressing tumors; KD: SDH5-deficient tumors; BLI: bioluminescent imaging).

To further verify the effect of SDH5 on radiosensitivity in vivo, two mouse models (orthotopic mice bearing lung cancer and SDH5 gene knockout mice) were established. In the orthotopic model, the lungs of male nude mice (6-8 weeks of age; n=3 per group) were exposed, and human lung cancer cells (A549) with SDH5 deficiency or con-expression were injected. Stable luciferase activity was confirmed in each subline to ensure equal levels before the injection. Radiological treatment was applied after successful tumor formation in the lungs of the mice. The mice were exposed to 0 or 20 Gy radiation. The SDH5-deficient tumors showed higher radiation sensitivity and smaller volumes (Figure 4A-B).

To further explore the underlying mechanisms, we examined RT-related proteins known to regulate DNA damage and apoptosis. As shown in Figure 4C-D and Figure S3, the SDH5-deficient tumors exhibited an increased level of p53, which is the most extensively studied tumor suppressor. Moreover, we observed an upregulation of γ-H2AX, which is a biomarker for DNA double-strand breaks (DSBs) 22, 23, and activated apoptosis pathways. The increased DSBs and apoptosis may explain the increased sensitivity to RT.

To further verify our conclusion, we used SDH5 KO mice. The KO strategy has been reported in our previous study 20. SDH5^(+/+)^ wild-type mice and SDH5^(-/-)^ knockout mice received lung radiation at a dose of 0 or 15 Gy/10F. Consistent with the data obtained from the tumor-bearing mouse models, γ-H2AX and p53 were upregulated, and the apoptosis pathways were activated in the SDH5^(-/-)^ knockout mice (Figure 4E-F), suggesting that the loss of SDH5 expression increased radiosensitivity in lung epithelial cells.

In response to irradiation, mammalian cells activate the cell cycle checkpoint, which may help prevent cell division and provide the necessary time for DNA damage repair or lead to apoptosis before DNA is repaired. p53-dependent apoptosis plays an important role in RT for tumors. Taken together, these data indicate that SDH5 deficiency may contribute to radiation sensitivity by increasing DNA damage and apoptosis in a p53-dependent manner.

### SDH5 negatively regulates p53 expression in multiple p53 wild-type cell lines

**Figure 5:**
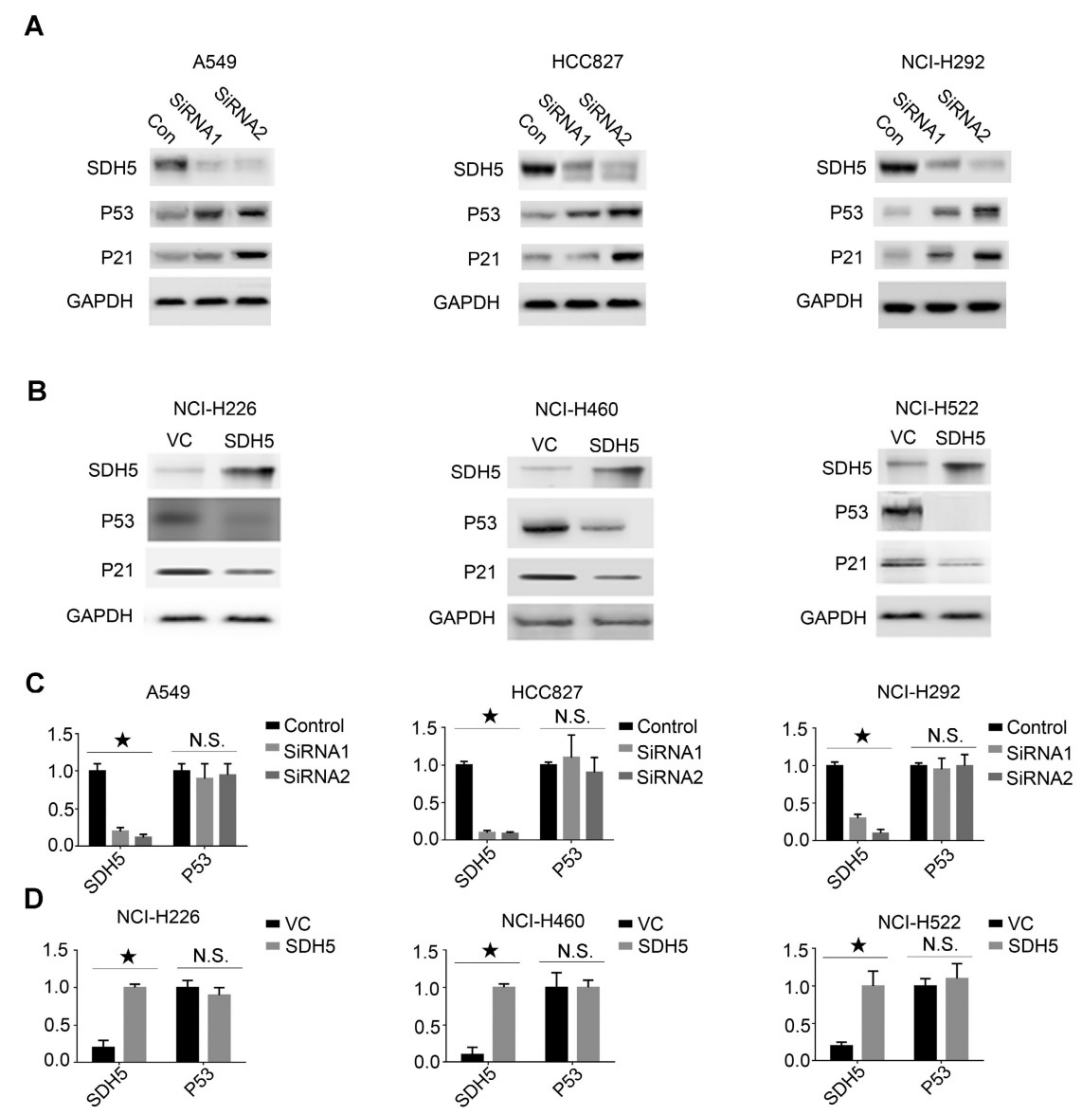
** SDH5 negatively regulates p53 expression at the protein level but not the mRNA level in multiple p53 wild-type cell lines. (A)** Immunoblotting analysis of the indicated proteins in A549, HCC827 and NCI-H292 cells after SDH5-SiRNA treatment. **(B)** Immunoblotting analysis of the indicated proteins in NCI-H226, NCI-H460 and NCI-H522 cells after SDH5 plasmid transfection. **(C)** mRNA levels of SDH5 and p53 in the indicated human lung cancer cells after SDH5-SiRNA treatment (★P<0.05; n.s.=no significance). **(D)** mRNA levels of SDH5 and p53 in the indicated human lung cancer cells after SDH5 plasmid transfection (★P<0.05; n.s.=no significance). The data are presented as the mean ± s.d. GAPDH was used as a loading control for the immunoblotting analysis.

Our in vivo data suggest that SDH5 deficiency may upregulate p53 expression and ultimately contribute to radiosensitivity by increasing DNA damage and apoptosis in vivo. To further explore the relationship between SDH5 and p53, we conducted an in vitro cell experiment and examined expression of the SDH5 protein in the following p53 wild-type lung cancer cell lines: A549, HCC827, and NCI-H292 cells expressing high levels of SDH5 and H226, NCI-H460, and H522 cells expressing low levels of SDH5. When SDH5 was knocked down by SDH5-SiRNA, the p53 protein and its target gene p21 were upregulated (Figure 5A). In contrast, when transfected with SDH5, the levels of the p53 and p21 proteins were obviously decreased (Figure 5B). However, the mRNA level of p53 did not significantly change regardless of the changes in SDH5 expression (Figure 5C-D). These data strongly suggest that SDH5 can negatively regulate expression of the wild-type p53 protein, which may occur through posttranslational modification.

### SDH5 directly binds the p53 proline-rich domain (PRD), promotes p53 phosphorylation and increases its nuclear accumulation in vitro

**Figure 6:**
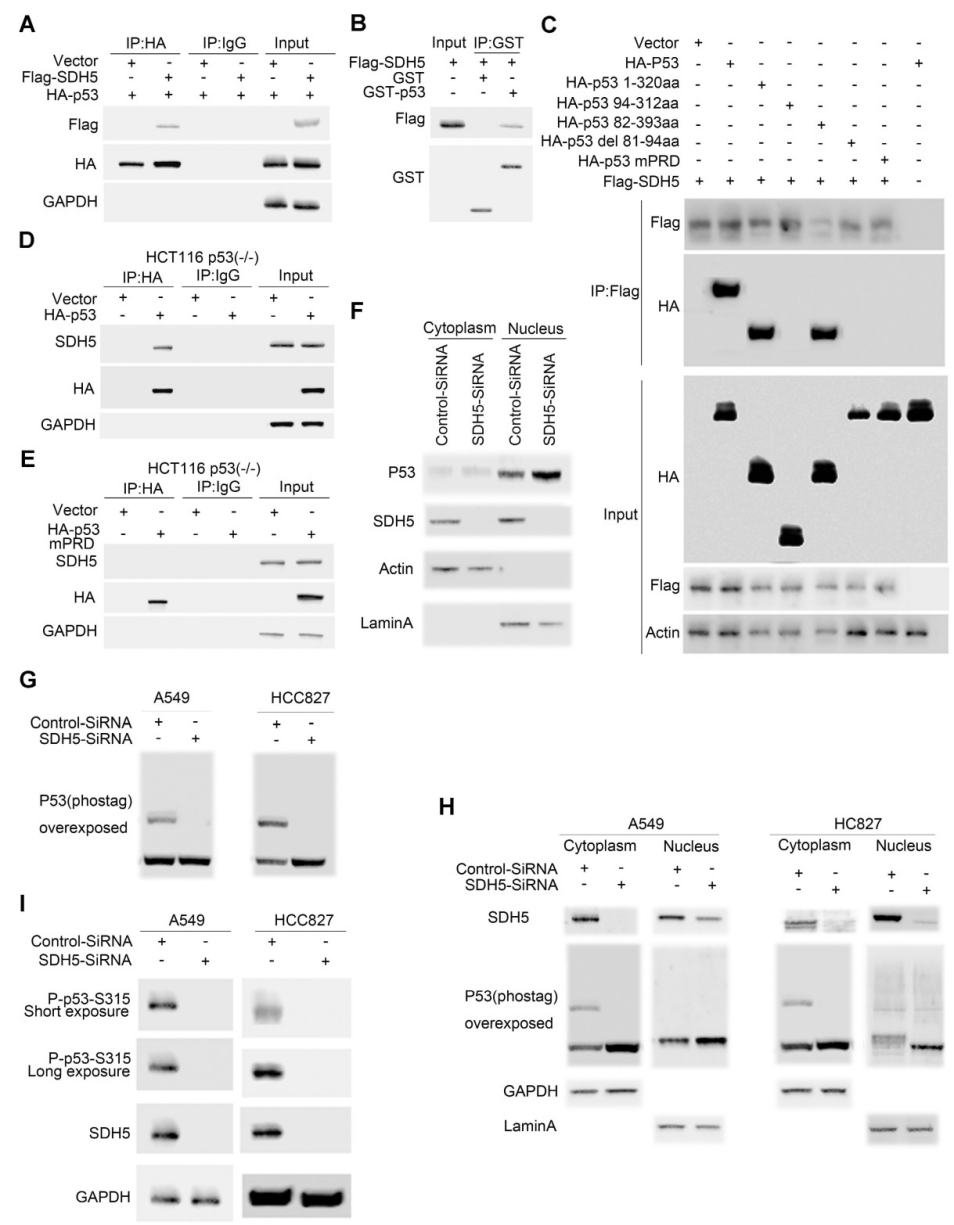
** SDH5 directly binds the p53 proline-rich domain, and SDH5 deficiency reduces p53 phosphorylation at Ser315. (A)** Co-IP assay revealed associations between the SDH5 and p53 proteins in HEK293T cell lines. **(B)** Beads coated with GST or GST-p53 fusion proteins were incubated with the Flag-SDH5 protein overnight. The GST pulldown was immunoblotted with the indicated antibodies. **(C)** IP-IB analysis of direct binding of p53 and its mutants to SDH5 in HEK293T cells. **(D)** and **(E)** IP-IB analysis of the binding of p53^WT^ and p53^mPRD^ to SDH5 in HCT116 p53^(-/-)^ cells. **(F)** IB analysis of p53 in the nuclear fraction and cytosol of cells after SDH5-SiRNA treatment for 72 h. **(G)** Phos-tag IB analysis of p53 phosphorylation in cells treated with SDH5-SiRNA for 72 h. **(H)** Phos-tag IB analysis of p53 phosphorylation in the nuclear fraction and cytosol of cells treated with SDH5-SiRNA for 72 h. **(I)** SDH5 knockdown reduced p53 phosphorylation at Ser315 in vitro. Cell lysates from A549 and HCC827 cells transfected with Control-SiRNA or SDH5-SiRNA were analyzed by Western blotting using the indicated antibodies. Actin, lamin A and GAPDH were measured as loading controls (IB: immunoblotting analysis; p53^WT^: wild-type p53; p53 ^mPRD^: p53 proline-rich domain mutation).

Accumulation of p53 activates its target genes and contributes to an SDH5 deficiency-dependent increase in radiosensitivity. To elucidate how SDH5 regulates the p53 protein and controls p53 function, we examined whether SDH5 could directly interact with p53 in cells. As expected, exogenously expressed SDH5 coimmunoprecipitated with p53 (Figure 6A). Importantly, we found that recombinant GST-tagged p53 immunoprecipitated with ectopically expressed SDH5 (Figure 6B), indicating that SDH5 directly interacts with p53 in vitro. To determine which region of p53 is involved in SDH5 binding, we generated a series of p53 mutations, including p53 1-320 aa (N-terminal 320 aa), p53 94-312 aa (aa 94-312), p53 82-393 aa (aa 82-393), p53 del81-94 aa (deletion of aa 81-94), and p53 mPRD (11 Ps converted to As in the proline-rich domain [PRD]). SDH5 binding p53 1-320 aa and p53 82-393 aa was comparable to its binding WT p53, but SDH5 failed to bind p53 94-312 aa, indicating that SDH5 may bind p53 82-94 aa. Moreover, deletion of the PRD (p53 del 81-94 aa) and p53 mPRD prevented binding SDH5. Thus, SDH5 directly binds the PRD in p53, and most, if not all, of the PRD sequence is involved in SDH5 binding (Figure 6C). We confirmed this finding in HCT116 (p53^-/-^) cells by immunoprecipitation of recombinant p53 with endogenously expressed SDH5, and the PRD mutant p53 protein failed to bind SDH5 (Figure 6D-E). Collectively, these findings suggest that SDH5 can directly interact with p53 and bind the PRD in vitro.

p53 is stabilized and activated and subsequently accumulates in the nucleus, where it induces the transactivation of many downstream target genes involved in cell cycle arrest, apoptosis, DNA repair, and senescence. To understand how SDH5 deficiency activates p53, we examined the subcellular distribution of p53 in A549 cells. SDH5 siRNA knocked down both cytosolic and nuclear SDH5, and p53 nuclear accumulation increased (Figure 6F). p53 accumulation in the nucleus triggers the gene transcription of its downstream targets. Since p53 phosphorylation has been reported to be associated with its nuclear distribution, we speculated that SDH5 might also affect this process. In both the A549 and HCC827 cell lines, SDH5 knockdown reduced p53 phosphorylation (Figure 6G). By analyzing the nuclear fraction and cytosolic fraction separately, we observed that SDH5 knockdown reduced p53 phosphorylation mainly in the cytoplasm (Figure 6H). Moreover, the phosphorylation of Ser315 was measured, and SDH5 knockdown reduced Ser315 phosphorylation in both A549 and HCC827 cell lines (Figure 6I). Collectively, our data demonstrate that SDH5 knockdown reduces p53 phosphorylation in the cytoplasm and increases its nuclear accumulation.

### SDH5 regulates p53 stability via the ubiquitin/proteasome pathway

**Figure 7:**
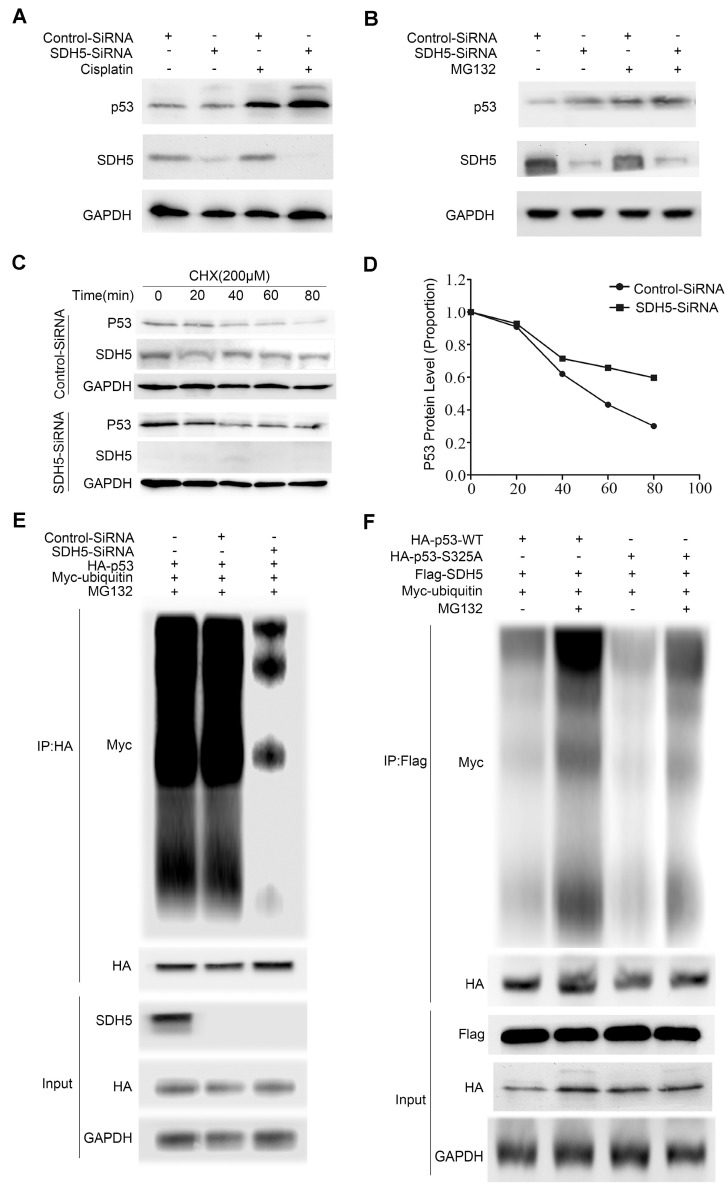
** SDH5 regulates p53 stability via the ubiquitin/proteasome pathway. (A)** A549 cells were transfected with SDH5-SiRNA or Control-SiRNA. After forty-eight hours, the cells were treated with 10 µM cisplatin or vehicle. p53 and SDH5 protein levels were determined by Western blotting. **(B)** A549 cells were transfected with SDH5-SiRNA or Control-SiRNA. After 48 h, the cells were treated with 10 µM MG132 (proteasome inhibitor) or vehicle and harvested after 2 h. p53 and SDH5 protein levels were determined by Western blotting. **(C)** and **(D)** After transfection with SDH5-SiRNA or Control-SiRNA for 48 h, the cells were treated with 100 µM cycloheximide (protein biosynthesis inhibitor) and harvested at different times. The levels of p53 and SDH5 were measured by Western blotting. ImageJ was used to quantify the p53 density, followed by normalization based on the p53 levels with the level at time point zero set as 1. **(E)** A549 cells were transfected with the indicated plasmids after treatment with SDH5-SiRNA for 24 h. The cells were harvested after treatment with 10 µM MG132 for 4 h. Then, the samples were subjected to immunoprecipitation and immunoblotted with the indicated antibodies. **(F)** A549 cells were transfected with the indicated constructs for 24 h and collected after treatment with MG132 (10 μM) for 4 h. The samples were subjected to immunoprecipitation and analyzed by Western blotting using the indicated antibodies. GAPDH was used as a loading control.

Considering that SDH5 negatively regulates p53 accumulation and that p53 is a labile protein degraded by the proteasome, we further investigated whether SDH5 regulates p53 stability. First, to confirm the effect of SDH5 on p53 signaling, we depleted SDH5 with or without cisplatin treatment. Cisplatin can cause DNA damage and subsequently activate p53 signaling. As shown in Figure 7A, SDH5 depletion increased p53 expression, and cisplatin-induced p53 levels were further enhanced. Additionally, as previously mentioned, SDH5 depletion did not increase expression of p53 mRNA (Figure 5C-D). These results suggest that SDH5 regulates p53 expression through posttranslational modifications. Furthermore, following treatment with the proteasome inhibitor MG132, SDH5 knockdown cells did not exhibit increased expression of p53 (Figure 7B). Finally, decreased expression of SDH5 increased the half-life of endogenous p53 (Figure 7C-D). We also performed ubiquitination assays and observed that p53 polyubiquitination was decreased when SDH5 was knocked down (Figure 7E), indicating that p53 degradation promoted by SDH5 is ubiquitination dependent. Since the stability of p53 is always related to its phosphorylation, we speculated that SDH5-dependent phosphorylation of p53 at Ser315 also leads to its degradation. As shown in Figure 7F, the S315A mutant displayed significantly reduced p53 ubiquitination compared to wild-type p53 in cells with ectopic expression of SDH5. These data suggest that SDH5 can regulate the stability of p53 in a phosphorylation-dependent manner.

### p53 mediated the biological effects of SDH5 on radiosensitivity

**Figure 8:**
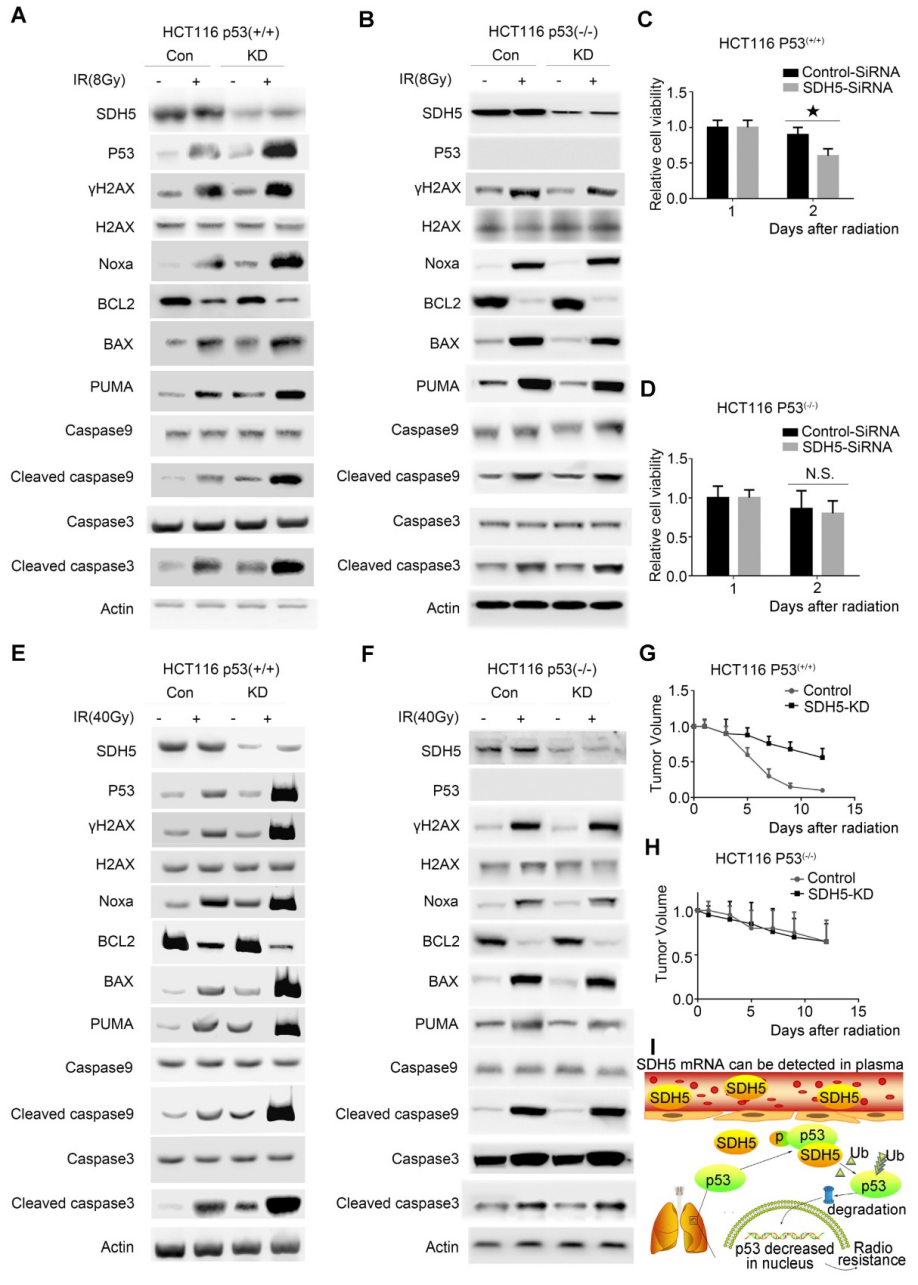
** p53-dependent apoptosis pathways mediated the biological effects of SDH5 on radiosensitivity. (A)**-**(D)** Measurement of cell viability and IB analysis of the indicated proteins in HCT116 p53^(+/+)^ and HCT116 p53^(-/-)^ cells transfected with SDH5-SiRNA or Control-SiRNA after 8 Gy radiation, followed by normalization based on cell viability with the level on the first day set as 1 (★P<0.05; n.s.=no significance). **(E)-(H)** KD (SDH5 knockdown) or Con (SDH5 con-expressing) HCT116 p53^(+/+)^ and HCT116 p53^(-/-)^ cells were subcutaneously injected into athymic nude mice and subjected to 40 Gy radiation (n=3 per group). Measurement of tumor growth and IB analysis of the indicated proteins in the tumors from athymic nude mice, followed by normalization based on the tumor volume with the level at time point zero set as 1. **(I)** The proposed model shows that SDH5 can be detected in plasma and tumor tissue and that SDH5 depletion enhances radiosensitivity by reducing p53 phosphorylation and stability in non-small cell lung cancer. SDH5 can bind the wild-type p53 protein via the proline-rich domain. Loss of SDH5 prevents p53 phosphorylation and promotes its degradation via the ubiquitin/proteasome pathway, leading to p53 accumulation in the nucleus, which ultimately enhances radiosensitivity in non-small cell lung cancer. The data are presented as the mean ± s.d. Actin was used as a loading control for the IB analysis (IB: immunoblotting analysis).

To confirm the functional roles of p53 in enhancing radiosensitivity caused by SDH5 knockdown, HCT116 p53^(+/+)^ and HCT116 p53^(-/-)^ cells were transfected with siRNAs targeting SDH5. As shown in Figure 8A-D, p53 deficiency partially rescued the defects in cell proliferation after RT in SDH5-depleted cells by inhibiting apoptosis pathways. Furthermore, consistent with our in vitro data, tumor growth was partially reversed when p53 and SDH5 were co-depleted (Figure 8E-H). Altogether, these data suggest that SDH5 exerts its functions mainly via its ability to inhibit p53-mediated apoptosis pathways.

## Discussion

Predicting the RT response is very important for clinical diagnosis, and p53 plays a critical role in predicting this response 8, 24. However, due to its very short half-life in tumor tissue, p53 is difficult to detect by IHC 25, rendering it a poor predictor of cancer. In this study, we found that SDH5 regulated the response to RT by interacting with p53 (Figure 1G-J) and that expression of SDH5 could be directly detected in tumors (Figure 1A-C), suggesting that SDH5 may be a suitable marker for predicting radiosensitivity. Furthermore, our results show that expression of SDH5 in plasma can be directly measured by qRT-PCR and that these levels are positively correlated with the expression level in the tumor (Figure 1D-F). This evidence provides new insight into a possible approach for predicting the RT response in patients who are unable to tolerate the biopsy process.

Numerous studies have reported that tumor radiosensitivity is associated with histological classification and molecular biological characteristics 26, 27. Lung biopsy remains the gold standard for histological classification because it allows a direct measurement of lung tissue 28. However, this procedure has limitations, including invasiveness, sampling error, and complications 9. As an invasive method, it is impossible to perform dynamic observation in tumors to evaluate the molecular biology characteristics. These reasons prompted us to explore alternative, noninvasive methods. The ideal noninvasive method should be simple, readily available, inexpensive, reliable, and accurate. Circulating cell-free nucleic acids, which mainly originate from apoptotic cells or spontaneous secretion, usually interact with proteins and lipids to form a complex to protect against nuclease degradation 29-31. Circulating cell-free nucleic acids, but not the target tissue, may be used as cancer biomarkers, as this approach is convenient and noninvasive. Furthermore, this approach can be widely applied for cancer screening and monitoring treatment efficiency. Here, we report that SDH5 mRNA can be detected in plasma and tumors, suggesting it as a potential biomarker for cancer RT, and this approach may provide a suitable noninvasive alternative method for predicting tumor radiosensitivity.

p53 is the most important tumor suppressor gene and acts as a central regulator of the cellular response to different types of stress signals, such as DNA damage, hypoxia, and nucleotide depletion 32-34. Under these conditions, p53 is activated, accumulates in the nucleus, and induces various downstream cellular effects involved in apoptosis, DNA repair, senescence and cell cycle arrest 35, 36. The relationship between p53 and tumor radiosensitivity has been established. Investigators have reported that suppression of p53 can switch wild-type mouse embryo fibroblasts from radiation sensitive to radiation resistant 36. Similar conclusions were reached using transgenic mice carrying the c-myc oncogene 37. Additionally, we recently reported that CDK16 promotes radioresistance by phosphorylating and degrading p53 8. Furthermore, the critical role of p53 in RT is tissue specific and dose dependent. Tumors originating from different tissues show different responses (apoptosis or cell cycle arrest) to radiation 24. A lower dose of radiation induces cell cycle arrest to repair DNA damage, and a higher dose of radiation causes apoptosis 38. Therefore, p53 is a key marker for predicting radiosensitivity in cancer patients.

SDH5 has been reported to be associated with several types of cancer 17-19 and play essential roles in the flavination of succinate dehydrogenase. In our previous study, we demonstrated that loss of SDH5 can facilitate the EMT, leading to lung cancer metastasis via the glycogen synthase kinase 3β/β-catenin pathway 20. Here, we examine the role and underlying mechanism of SDH5 in the radiosensitivity of lung cancer. First, we initiated an analytical, observational, open, retrospective study (ChiCTR1800014878) involving 208 patients with stage III lung cancer and found that SDH5 could be detected in the plasma (Figure 1D-F). Interestingly, SDH5 expression was associated with PFS and OS in the patients receiving radiation, and the results obtained from plasma and tumor samples were consistent (Figure 1G-J). We confirmed this finding in animal experiments (Figure 4A-B). Thus, SDH5 may be useful as a good marker for predicting the RT response in lung cancer patients, especially in those who cannot tolerate biopsy. Subsequently, we explored the possible mechanisms by which SDH5 affects the RT response. Ionizing radiation triggers a spectrum of responses in tumor cells, including DNA damage, apoptosis, necrosis, and senescence 39, 40. During RT, radiation-induced DNA damage can lead to cell death by activating different cellular events, and DNA damage can cause cell death by activating downstream signaling pathways, such as p53, MAPKs and AKT. We investigated expression and phosphorylation of DNA damage-related proteins, including histones and p53, in treated cells, as the p53 status has often been considered central to the radiation response of tumor cells. Our results demonstrated that SDH5 depletion increased expression of γH2AX and p53 and activation of a series of apoptosis-related proteins after radiation (Figure 2C-D; Figure 4D-F; Figure 8A-C). As a tumor suppressor gene and an important nuclear transcription factor, the main function of p53 is to monitor the integrity of cellular genomic DNA. After ionizing radiation, p53 first promotes cell cycle arrest and DNA damage repair. If this repair cannot be completed, p53 can regulate expression of a series of apoptosis-related proteins, such as Bax, PUMA, and Bcl2, to induce cell apoptosis, remove damaged cells and prevent carcinogenesis. To explore whether p53 is required for the regulation of SDH5 in apoptosis after radiation, we conducted in vitro experiments and found that SDH5 negatively regulates p53 expression in different wild-type cell lines. More importantly, our study showed that SDH5 directly interacts with p53 by binding its PRD (Figure 6), leading to p53 phosphorylation at Ser315 to promote p53 degradation via the ubiquitin/proteasome machinery (Figure 6 and Figure 7E-F). As expected, p53 depletion rescued these phenotypes caused by SDH5 deficiency (Figure 8), suggesting that p53 is the major downstream effector of SDH5, which functions to enhance radiosensitivity by regulating apoptosis pathways in lung cancer.

In summary, we identified that SDH5 is a novel regulator of p53 and that loss of SDH5 enhances radiosensitivity by reducing p53 phosphorylation and delaying p53 degradation in lung cancer, as proposed in Figure 8I. SDH5-dependent p53 activation leads to increased apoptosis after radiation. Most importantly, the SDH5 expression levels in plasma and tumors were positively correlated (rho=0.894, P<0.001), and lung cancer patients with lower expression of SDH5 had longer PFS (average median PFS 30.0 versus 15.0 months, P<0.001) and OS (average median OS 38.0 versus 25.0 months, P<0.001) after radiation. We believe that SDH5 is a promising biomarker for predicting prognosis and the RT response in patients with lung cancer, especially in old or weak patients who cannot tolerate biopsy. Overall, this factor is a promising target for lung cancer RT.

## Supplementary Material

Supplementary figures.

## Figures and Tables

**Table 1:** Baseline demographic and clinical characteristics in this study.

Characteristic	N	%
**Age**		
<60	93	44.71
≥60	115	55.29
**Sex**		
Male	122	58.65
Female	86	41.35
**Stage**		
IIIA	32	15.38
IIIB	176	84.62
**Histological type**		
Adenocarcinoma	51	24.52
Squamous carcinoma	157	75.48
**Treatment**		
Radiotherapy plus TP*	51	24.52
Radiotherapy plus EP**	157	75.48

*TP: Paclitaxel combined with cisplatin **EP: Etoposide combined with cisplatin

## Abbreviations

SDH5: succinate dehydrogenase 5
NSCLC: non-small cell lung cancer
RT: radiotherapy
CEA: carcinoembryonic antigen
NSE: neuron-specific enolase
SCC-Ag: squamous cell carcinoma antigen
CT: computed tomography
PFS: progression-free survival
OS: overall survival
IHC: immunohistochemistry
RECIST: response evaluation criteria in solid tumors
GTV: gross tumor volume
DNA-PK: DNA-dependent protein kinase
NHEJ: nonhomologous end joining
DSBs: DNA double-strand breaks
