# Supplementary material for: SDH5 Depletion Enhances Radiosensitivity by Regulating p53: A New Method for Noninvasive Prediction of Radiotherapy Response
Source: Theranostics. 2019 Aug 14;9(22):6380–95. doi: 10.7150/thno.34443 (PMC6771232; doi:10.7150/thno.34443)
Supplement: Supplementary file 1 — Supplementary figures. [file thnov09p6380s1.pdf]

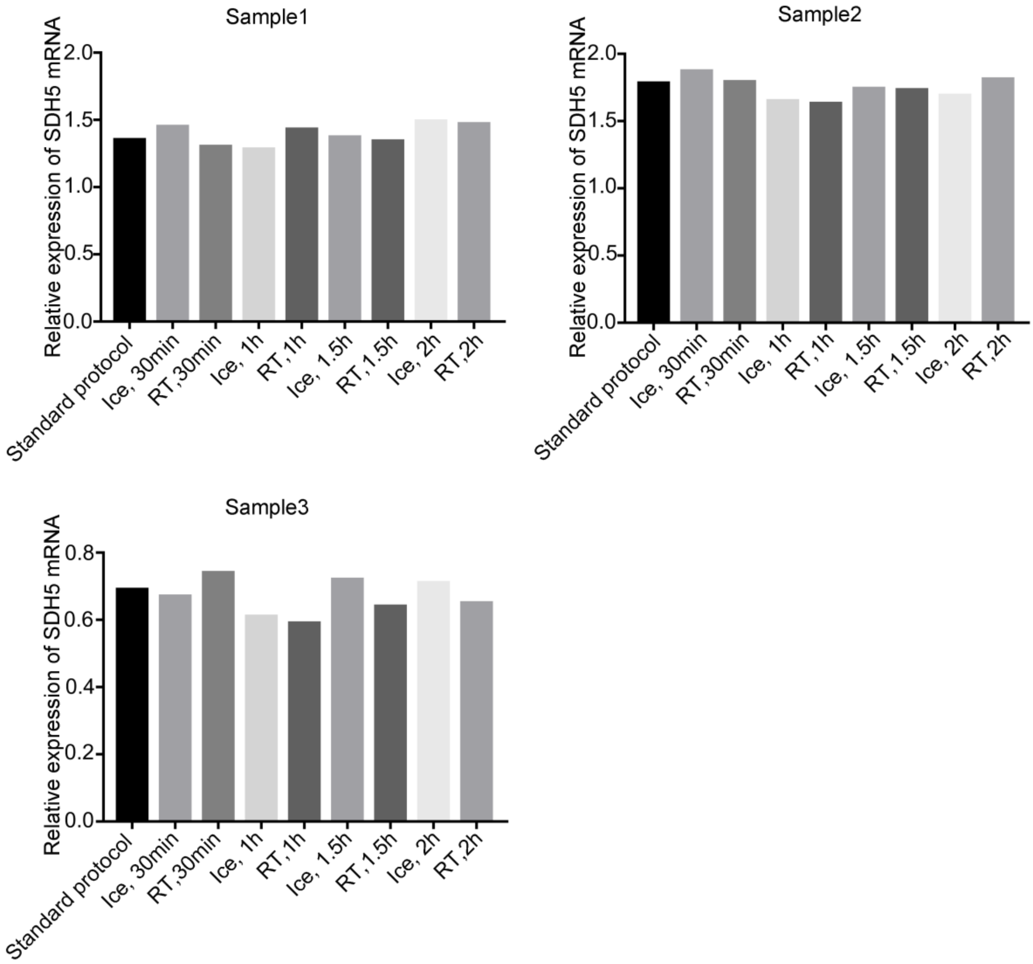

Figure S1. Validation of the stability of plasma mRNA. Plasma mRNA levels of SDH5 were stable after being subjected to various conditions.

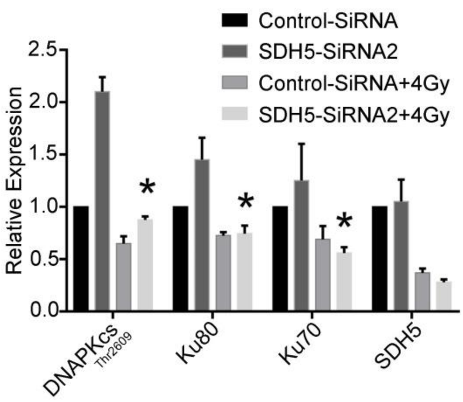

Figure S2. Quantitative detection of indicated peoteins in A549 after 4 Gy radiation. Histograms of quantifcation expression of indicated proteins based on the mean of three independent experiments; Actin was used as the endogenous control. Error bar shows the standard error.

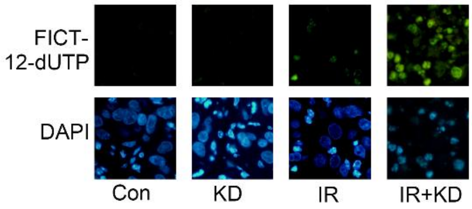

Figure S3. Apoptosis of SDH5-deficient tumors after radiation. Effects of SDH5 deficiency on radiation-induced apoptosis in tumor tissues were investigated by the Tunnel assay. Apoptotic cells were stained with FITC-12-dUTP (green). Cell nucleus were stained with DAPI (blue).
